# Interhemispheric modulations of motor outputs by the rostral and caudal forelimb areas in rats

**DOI:** 10.1152/jn.00591.2019

**Published:** 2020-03-04

**Authors:** Boris Touvykine, Guillaume Elgbeili, Stephan Quessy, Numa Dancause

**Affiliations:** ^1^Département de Neurosciences, Faculté de Médecine, Université de Montréal, Québec, Canada; ^2^Psychosocial Research Division, Douglas Institute Research Centre, Verdun, Québec, Canada

**Keywords:** cortical network, forelimb, hand, motor outputs, premotor cortex, TMS

## Abstract

In rats, forelimb movements are evoked from two cortical regions, the caudal and rostral forelimb areas (CFA and RFA, respectively). These areas are densely interconnected and RFA induces complex and powerful modulations of CFA outputs. CFA and RFA also have interhemispheric connections, and these areas from both hemispheres send projections to common targets along the motor axis, providing multiple potential sites of interactions for movement production. Our objective was to characterize how CFA and RFA in one hemisphere can modulate motor outputs of the opposite hemisphere. To do so, we used paired-pulse protocols with intracortical microstimulation techniques (ICMS), while recording electromyographic (EMG) activity of forelimb muscles in sedated rats. A subthreshold conditioning stimulation was applied in either CFA or RFA in one hemisphere simultaneously or before a suprathreshold test stimulation in either CFA or RFA in the opposite hemisphere. Both CFA and RFA tended to facilitate motor outputs with short (0–2.5 ms) or long (20–35 ms) delays between the conditioning and test stimuli. In contrast, they tended to inhibit motor outputs with intermediate delays, in particular 10 ms. When comparing the two areas, we found that facilitatory effects from RFA were more frequent and powerful than the ones from CFA. In contrast, inhibitory effects from CFA on its homolog were more frequent and powerful than the ones from RFA. Our results demonstrate that interhemispheric modulations from CFA and RFA share some similarities but also have clear differences that could sustain specific functions these cortical areas carry for the generation of forelimb movements.

**NEW & NOTEWORTHY** We show that caudal and rostral forelimb areas (CFA and RFA) have distinct effects on motor outputs from the opposite hemisphere, supporting that they are distinct nodes in the motor network of rats. However, the pattern of interhemispheric modulations from RFA has no clear equivalent among premotor areas in nonhuman primates, suggesting they contribute differently to the generation of ipsilateral hand movements. Understanding these interspecies differences is important given the common use of rodent models in motor control and recovery studies.

## INTRODUCTION

In nonhuman primates (NHPs), the largest proportion of the corticospinal tract originates from the primary motor cortex (M1) (Dum and Strick 1991). In addition, there are six premotor cortical areas that send projections to the spinal cord and to M1 (Dum and Strick 2002). Electrical stimulations in M1 and premotor areas can evoke electromyographic (EMG) responses in forelimb muscles (Boudrias et al. 2010a; Boudrias et al. 2010b; Park et al. 2004). In contrast, although rats are capable of dexterous movements (Whishaw 1996), responses in forelimb muscles are evoked from only two cortical areas (Neafsey et al. 1986; Neafsey and Sievert 1982).

The larger of these two motor regions, the caudal forelimb area (CFA), is the origin of the majority of corticospinal neurons and is located predominantly in the lateral agranular cortex (Neafsey et al. 1986; Rouiller et al. 1993). CFA projects to the ventrolateral thalamic nucleus, a pathway shared by M1 in NHPs (Rouiller et al. 1998). Based on these features, CFA is generally considered to be a homolog of M1. The smaller forelimb motor region is rostral to CFA, in the medial agranular cortex and is consequently referred to as the rostral forelimb area (RFA) (Neafsey et al. 1986; Neafsey and Sievert 1982). Like CFA, RFA sends projections to the spinal cord (Neafsey et al. 1986; Rouiller et al. 1993), and most RFA neurons discharge prior and during contralateral reaching and grasping, supporting its role in the preparation and execution of contralateral forelimb movements (Hyland 1998; Zhuravin and Bures 1988, 1989). However, there are discrepancies between the pattern of anatomical projections of CFA and RFA. First, corticospinal projections from RFA are sparser than from CFA (Neafsey et al. 1986; Starkey et al. 2012). Second, corticothalamic projections from RFA predominantly target the ventromedial thalamic nucleus (Rouiller et al. 1993). Third, unlike CFA, RFA is interconnected with the insular cortex, a pathway present for premotor areas but not M1 of NHPs (Jürgens 1984; Matelli et al. 1986; Stepniewska et al. 1993). Together, this pattern of connections suggests that RFA could be an homolog of a premotor area in NHPs. Moreover, much like a premotor area, RFA sends projections to CFA (Rouiller et al. 1993) and can exert powerful intrahemispheric modulations of CFA outputs (Deffeyes et al. 2015).

Similar to premotor areas and M1 in NHPs (Dancause et al. 2007; Marconi et al. 2003; Rouiller et al. 1994), RFA and CFA are interconnected across the two hemispheres and form a complex bihemispheric network (Rouiller et al. 1993). Moreover, RFA and CFA from both hemispheres send projections to common targets along the motor axis, providing multiple potential sites of interactions for their outputs. This anatomical substrate may allow the involvement of RFA and CFA in the control of the ipsilateral arm and hand. Not surprisingly, some neurons in these regions are modulated during ipsilateral movements (Dolbakyan et al. 1977; Soma et al. 2017) and motor cortex lesions induce deficits in the ipsilateral forelimb (Gonzalez et al. 2004; Price and Fowler 1981). Moreover, inactivation or permanent cortical injuries affecting motor cortex induce changes in the size of CFA and RFA in the opposite hemisphere (Maggiolini et al. 2008; Touvykine et al. 2016), suggesting the presence of reciprocal interhemispheric influences. Yet, the modulatory impact of CFA and RFA on the production of motor outputs from opposite hemisphere has not been investigated. These data would reveal similarities and differences between CFA and RFA and help us understand how these areas can contribute to the production of movements of the ipsilateral arm. Moreover, they would allow us to compare patterns of interhemispheric modulations of RFA to the ones of premotor areas in NHPs (Côté et al. 2017, 2020; Quessy et al. 2016). Therefore, we sought to characterize output properties of CFA and RFA and then studied how these outputs are modulated by conditioning stimulations delivered in forelimb motor areas of the opposite hemisphere. In the present set of experiments, we focused on the modulation from CFA and RFA on their respective homolog in the opposite hemisphere. In addition, considering that RFA has been suggested to be on a higher hierarchical level than CFA (Rouiller et al. 1993), we also studied modulatory effects from RFA on motor outputs of CFA. We discuss how these effects compare with the pattern of interhemispheric modulation between premotor areas and M1 in NHPs (Côté et al. 2017, 2020; Quessy et al. 2016).

## MATERIALS AND METHODS

### 

#### Subjects.

Our experimental protocol followed the guidelines of the Canadian Council on Animal Care and was approved by the Comité de Déontologie de l’Expérimentation sur les Animaux of the Université de Montréal. Thirteen single housed adult Sprague-Dawley rats weighing between 307 g and 480 g, with unrestricted but identical enrichment and food and water ad libitum, were used in our experiments (mean = 380 g). All animals were at least 3½ months of age with the oldest animal being 5 mo old at the time of the experiment (mean age ~4 mo). Females were chosen to keep in line with our previous work (Deffeyes et al. 2015; Mansoori et al. 2014; Touvykine et al. 2016). To the best of our knowledge, there are no reported differences between male and female rats in regard to the pattern of anatomical projections from either CFA or RFA. Accordingly, modulatory effects should be consistent across sexes, although this will have to be tested experimentally. We collected data to study the modulatory effect of CFA on the outputs of CFA in the opposite hemisphere in 7 out of 13 animals (Table 1). The modulatory effects of RFA on the outputs of CFA in the opposite hemisphere were collected in five rats. Finally, the effects of RFA on the outputs of RFA in the opposite hemisphere were studied in 7 out of the 13 rats.

**Table 1.** A summary of the number of ICMS sites required to define the border and the number of protocols collected per animal

			Number of Protocols					
	Number of ICMS Sites to Define Border		CFA-CFA		RFA-CFA		RFA-RFA	
Rat ID	Right	Left	T_stim_ Right	T_stim_ Left	T_stim_ Right	T_stim_ Left	T_stim_ Right	T_stim_ Left
002	5	4	0	0	2	0	0	2
004	5	4	0	0	0	0	2	0
049	6	6	3	0	0	0	0	0
050	4	7	1	0	0	0	0	2
082	3	12	4	0	0	0	2	0
083	3	4	4	0	0	0	3	0
086	3	5	0	2	0	0	0	2
089	7	4	0	5	0	0	0	0
099	37	11	0	0	0	3	0	0
100	11	9	0	1	2	1	0	0
119	25	21	0	0	4	0	0	0
126	5	4	0	0	4	1	0	0
127	17	14	0	0	0	0	1	0

Number of intracortical microstimulation techniques (ICMS) sites report the number cortical that needed to be inspected with ICMS trains to define the border between caudal and rostral forelimb areas (CFA and RFA) in each hemisphere. Number of protocols report the number of protocols collected for each type of modulatory effect studied (i.e., CFA-CFA, RFA-CFA, and RFA-RFA) and in which hemisphere the test stimulus (T_stim_) was located.

#### Surgical procedures.

All surgical procedures were performed as part of an aseptic, nonsterile, terminal experiment (Deffeyes et al. 2015; Touvykine et al. 2016). Anaesthesia was induced with a single dose of ketamine hydrochloride via intraperitoneal injection (80 mg/kg). The animals were transitioned to ~2% isoflurane general anesthesia (Furane; Baxter) in 100% oxygen and remained under general anesthesia for the duration of the surgical procedures. They received a dose of dexamethasone (1 mg/kg) intramuscularly and mannitol (~3,000 mg/kg) intraperitoneally to prevent the inflammation and swelling of the brain, respectively. Subcutaneous injections of physiological saline (2 ml) were delivered every 2 h to prevent dehydration. Body temperature was monitored continuously via an anal probe and kept to ~36.0°C with a homeothermic blanket system (Harvard Apparatus, Holliston, MA).

Multistranded microwires (Cooner Wire, Chatsworth, CA) were implanted intramuscularly to record electromyographic (EMG) signals. In the forearm, we implanted the extensor digitorum communis, a wrist extensor (WE), and the palmaris longus, a wrist flexor (WF). In the arm, we implanted the biceps brachii, an elbow flexor (EF) and the tricep brachii. Finally, we implanted the spinodeltoid on the back. However, in offline analyses, few responses were observed in the tricep brachii and spinodeltoid. We therefore focused our analyses on WE, WF, and EF muscles, for which we had sufficient data. For each muscle, the accurate placement of the EMG wires was confirmed using electrical stimulations through the implanted wires and visual inspection of the evoked movements. To ensure the quality of implantation, movements had to be evoked through the EMG wires with stimulation intensities <300 µA. Following the implantation of EMG wires, the animal was positioned in a stereotaxic frame and bilateral craniectomies and durectomies were performed to expose forelimb motor areas of the two cerebral hemispheres. Craniectomies exposed the cortex from ~5 mm to −1.5 mm anteroposterior and 1.5 mm to 6 mm mediolateral in relation to bregma. Upon completion of the durectomies, the exposed cortex was covered in warm neutral mineral oil to prevent dehydration and was added as needed until the end of the procedure.

#### Localization of motor representations.

**Fig. 1.**
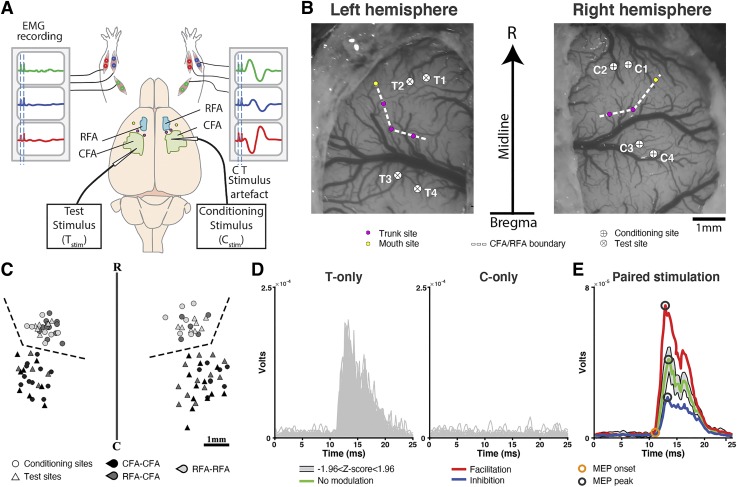
Experimental setup and location of cortical stimulation sites. *A*: schematic representation of the experimental setup. EMG signals from 3 forelimb muscles (WE, wrist extensor; WF: wrist flexor; EF, elbow flexor) implanted bilaterally were analyzed. For paired pulse stimulation protocols, the electrode delivering the conditioning stimulus (C_stim_) was positioned in either caudal or rostral forelimb areas (CFA or RFA) of 1 hemisphere. The electrode delivering the test stimulus (T_stim_) was positioned in either CFA or RFA of the opposite hemisphere. The stimulation intensity of the T_stim_ was suprathreshold and evoked EMG responses in the contralateral arm (right arm EMG recording). For the C_stim_, the intensity was subthreshold and did not evoke motor-evoked potentials (MEPs) in either arm (left arm EMG recording). *B*: example of bilateral craniotomy showing the location of the C_stim_ and T_stim_ electrodes in a rat. Note that the figure is an exact scaled reproduction of the position and orientation of the 2 craniotomies in relation to the skull midline (vertical arrow) and bregma (horizontal line on the arrow). Intracortical microstimulation techniques (ICMS) trains were first used to locate nonforelimb responses, in this case neck (purple dots) and mouth (yellow dots) movements, to define the border between RFA and CFA (white dashed line). In this animal, a total of 4 paired pulse protocols were conducted. In the first 2 protocols, the C_stim_ was delivered in RFA (C1 and C2) of 1 hemisphere and T_stim_ in RFA of the opposite hemisphere (T1 and T2). In the last 2 protocols, the C_stim_ was delivered in CFA (C3 and C4) of 1 hemisphere and T_stim_ in CFA of the opposite hemisphere (T3 and T4). *C*: schematic representation of all Test and Conditioning sites. Test (triangles) and Conditioning (circles) sites for CFA-CFA (black), RFA-CFA (dark gray), and RFA-RFA (light gray) protocols are aligned on the border (dashed line) between RFA and CFA. R, rostral; C, caudal. *D*: example of responses evoked in WE contralateral to T_stim_ with single pulses in the T_stim_ delivered alone (T-only; *n* = 100; *left*) and C_stim_ delivered alone (C-only; *n* = 100; *right*) conditions. EMG traces are rectified and smoothed. As expected, the T_stim_ evoked a clear MEP and the C_stim_ did not evoke any clear responses. *E*: to quantify the modulation of the MEP in the paired stimulation conditions, we first calculated a predictor (shaded gray area) based on the responses obtained in the T-only and C-only conditions shown in *D*. Responses evoked in the paired conditions were compared with this predictor. In this example, when the C_stim_ was delivered simultaneously with the T_stim_ [interstimulus interval (ISI)0; red trace], the amplitude of the MEP was significantly greater than the predictors (*Z*-score ≥ 1.96) and the effect was classified as facilitatory. When the C_stim_ was delivered 15 ms before the T_stim_ (ISI15; green trace), the amplitude of the MEP fell within the predictors and the effect was classified nonsignificant. Finally, when the C_stim_ was delivered 10 ms before the T_stim_ (ISI10; blue trace), the amplitude of the MEP was significantly smaller than the predictors (*Z*-score ≤ 1.96) and the effect was classified as inhibitory. Open circles on the traces show the response peak (black) and onset (orange) time that were used to calculate the amplitude of the responses.

At the end of the surgical procedures, isoflurane was turned off and deep sedation was maintained with intraperitoneal injections of ketamine hydrochloride (~3–5 mg/kg/10 min) (Touvykine et al. 2016). Before collection of electrophysiological data for the paired-pulse experiments, RFA and CFA were located using intracortical microstimulation (ICMS) trains delivered at 1 Hz and generated by RZ5 real-time processor [Tucker Davis Technologies (TDT), Alachua, FL]. Each train consisted of 13 cathodal 0.2-ms duration square pulses with 3.3-ms interpulse interval (Touvykine et al. 2016). For each cortical site tested, a glass-insulated tungsten microelectrode (~0.5-MΩ impedance; FHC, Bowdoin, ME) was lowered 1,500–1,600 µm below the cortical surface with a microdrive (model 2662; David Kopf Instruments, Tujunga, CA) mounted on a micromanipulator (David Kopf Instruments, Tujunga, CA). Trains were delivered with a constant current stimulus isolator (model B51-2; BAK, Mount Airy, MD), and the intensity was gradually increased to a maximum current intensity of 100 µA or until a clear movement was evoked. Movements were categorized as forelimb, neck, vibrissae, or mouth (jaw or tongue movements). To delineate RFA from CFA we characterized a strip of cortex between these two areas from which nonforelimb responses (typically neck movements) were evoked (Kleim et al. 1998; Neafsey et al. 1986; Touvykine et al. 2016). To further confirm the location of RFA, we explored the cortical territory laterally to this representation to confirm that mouth motor responses were evoked, as expected (Neafsey et al. 1986) (Fig. 1, *A* and *B*). An average of 9.1 ± 8.1 (means ± SD) cortical sites were required to define this border (minimum = 3; maximum = 37) (Table 1). Accordingly, cortical sites evoking forelimb movements located rostral to the strip of neck responses and medial to the mouth representation were considered to be in RFA. Forelimb responses caudal to the strip of neck motor responses were considered to be in CFA. After the border between CFA and RFA was established in both hemispheres, no further mapping was done on the experimental animals, and we proceeded to collect paired-pulse stimulation protocols.

#### Paired-pulse stimulations and EMG recording.

For the paired-pulse experiments, two glass-coated tungsten microelectrodes (~0.5 MΩ impedance; FHC, Bowdoin, ME) were positioned with two independent micromanipulators. In different protocols, the conditioning stimulation (C_stim_) electrode was either positioned in CFA or RFA of one hemisphere and the test stimulation (T_stim_) electrode was positioned in either CFA or RFA of the opposite hemisphere. We have conducted a total of 54 paired pulse protocols in 13 rats. Protocols were separated in three different types. First, in some protocols (*n* = 20) the C_stim_ was delivered in CFA and the T_stim_ in the opposite CFA to characterize the modulatory effects from CFA on motor outputs of its homolog (i.e., CFA-CFA protocols). In the second type of protocols (*n* = 18) the C_stim_ was delivered in RFA and the T_stim_ in CFA to characterize the modulatory effects from RFA on motor outputs of CFA in the opposite hemisphere (i.e., RFA-CFA protocols). In the third type of protocols (*n* = 16), the C_stim_ was delivered in RFA and the T_stim_ in RFA of the opposite hemisphere to characterize the modulatory effects from RFA on motor outputs of its homolog (i.e., RFA-RFA protocols).

For each cortical site included in these protocols, we first confirmed that it evoked clear forelimb movements in the arm contralateral to the electrodes using ICMS trains. Stimulations were then switched to single, 0.2-ms cathodal pulses delivered at 2 Hz. The current intensity was increased to a maximal intensity of 300 µA, while simultaneously looking at the EMG signals of all recorded muscles on a custom-built interface using OpenEx software (TDT, Alachua, FL). Once a motor-evoked potential (MEP) was identified in at least one of the muscles in the arm contralateral to the stimulation, the intensity was adjusted to establish the threshold value. This procedure was used for both the electrodes delivering the T_stim_ and C_stim_. The T_stim_ intensity was then set to 125% of the threshold value, and the C_stim_ intensity was set to 75% of threshold. For the T_stim_, if we found that the MEP was either too big or too small with 125% of the threshold value after the initiation of data collection of the protocol, data collection was interrupted. Stimulation intensity was adjusted to reestablish threshold stimulation intensity, and the protocol was reinitiated. For example, if we found that the single-pulse T_stim_ evoked twitches on a large proportion of trials, the stimulation was considered “too big” and the stimulation intensity was decreased to reestablish thresholds current intensity. This however did not occur in the present set of experiments. Alternatively, if after initiation of the protocol we observed that the single-pulse T_stim_ evoked responses in less than 50% of trials with visual inspection on the oscilloscope, the intensity was considered “too small” and the stimulation intensity was increased. This was necessary for 5.9% (*n* = 3) of T_stim_ sites included in the study. Having a large enough response with the T_stim_ was essential to ensure that the C_stim_ could either increase or decrease this response. If no MEPs were evoked with T_stim_ using the maximal stimulation intensity of 300 µA, the cortical site was discarded and the electrode moved to another location. For the C_stim_, if no response was observed using single pulses with the maximum intensity of 300 µA, the stimulation intensity was arbitrarily set to 225 µA (i.e., 75% of 300 µA). This was the case for 37.3% (*n* = 19) of C_stim_ sites included in the study.

Once the cortical sites were identified and stimulation intensities established, a paired-pulse stimulation protocol was initiated. The protocol included nine stimulation conditions: the T_stim_ delivered alone (T-only trials), the C_stim_ delivered alone (C-only trials), or both the C_stim_ and the T_stim_ delivered (paired stimulation) with one of seven different interstimulus intervals (ISIs). In the paired stimulation conditions, the C_stim_ and the T_stim_ were either delivered simultaneously (ISI0) or with the C_stim_ preceding the T_stim_ by 2.5 ms (ISI2.5), 5 ms (ISI5), 10 ms (ISI10), 15 ms (ISI15), 20 ms (ISI20), and 35 ms (ISI35).

During a protocol, the stimulation condition was randomized across trials, until a total of 100 trials were collected for each stimulation condition (total number of trials for each protocol = 900). All cortical sites in an animal were only used once. After data collection for a paired pulse protocol was completed, both electrodes were moved to new cortical locations, and all procedures were repeated. The selection of these additional cortical sites was random. We did not attempt to specifically select cortical sites based on corresponding stereotaxic locations or find comparable location within the motor representations (Fig. 1*C*). On average, 3.9 ± 1.6 (means ± SD) paired-pulse protocols were collected per animal (minimum = 1; maximum = 7; see Table 1). Data collection was stopped after ~5 h from the moment the cortex was exposed to ensure stable responsiveness of the preparation and avoid any potential effects of overstimulation.

We verified if the order of data collection (i.e., rank at which each site was tested within a given rat) had affected modulatory effects using a mixed linear model. In this model, the dependent variable was the *Z*-score, the fixed factors were the type of protocol (3 levels), the site rank (7 levels), and ISI (7 levels), and the random factors were site rank nested within rat (13 levels). We found that site rank was not a significant predictor of *Z*-scores (*P* = 0.79). Based on this analysis, it appears that order in which data was collected across cortical sites within an animal does not affect the pattern of modulation. This finding is in line with the lack of cumulative effects we have previously found when using similar protocols in both rats (Deffeyes et al. 2015) and monkeys (Côté et al. 2017, 2020; Quessy et al. 2016).

The EMG data were recorded with custom OpenEx software running on an RZ5 real-time processor (TDT, Alachua, FL). Each EMG channel was recorded at 4.9 kHz, and raw EMG data were stored for offline analysis.

#### EMG data analyses.

EMG analyses were conducted offline using custom-written MATLAB (version R2013a; Natick, MA) code. The continuous EMG data collected during the experiments were separated into individual trials for each condition and aligned to stimulation time stamps. The signal was full-wave rectified and smoothed using five-point moving average (window size = 1.02 ms), with no additional filtering. For each condition, the baseline was calculated from a 25-ms window before the first stimulus (−26 to −1 ms before the first stimulus timestamp). The motor-evoked potentials (MEPs) were calculated from a window of 3 to 30 ms after the end of the last stimulation timestamp. For each channel of recorded EMG, we first averaged all trials in the T-only condition and compared the average MEP to the average baseline activity for the same trials. MEPs with amplitudes >1 SD above baseline value were considered large enough to be either facilitated or inhibited by C_stim_ and kept for further analyses. Cases in which the MEP with the T-only condition smaller than 1 SD above baseline were excluded. Second, we verified that the C-only condition did not evoke MEPs in the muscles of the arm contralateral to the C_stim_ electrode. This validates the assumption of linear summation for the calculation of the predictor (see below) (Baker and Lemon 1995). Three protocols were removed due to such undesirable responses. Out of the 54 protocols, 3 had no significant MEP (>1 SD above baseline) in offline analyses and were rejected from further analyses. From the 51 remaining protocols, a total of 143 significant MEPs with the T-only condition in the arm contralateral to the T_stim_, and that has satisfied the criterion of being >1 SD, above the baseline were included in the study. These MEPs were used to characterize and compare the output properties of CFA and RFA (see below).

We then quantified the modulatory effect of the C_stim_ on each of the 143 significant MEPs in paired-pulse conditions. We first calculated a predictor using a modified bootstrapping procedure to generate a population of predicted responses that could be observed, if no interaction took place between the outputs from Test and Conditioning cortical sites (Quessy et al. 2016). To this end, we linearly summed all possible combinations of single T-only traces (*n* = 100) with single C-only traces (*n* = 100) to generate a population of 10,000 traces. It should also be noted that since we ensured that the C_stim_ was subthreshold, any resulting MEPs are largely driven by T-only responses. Next, we randomly drew 100 traces from the population of predicted traces and averaged them to create an average predicted MEP. For each of the average predicted MEPs we found the peak voltage by taking the maximum value between 8 and 23 ms after the T_stim_ and subtracting the voltage value at peak onset. Peak onset was found by performing a backward march that started at 10% of the peak maximum voltage value. The voltage value of each data point was compared with the one of the next point, moving back toward the stimulus onset. If the difference between the two points was <4% of the value of the first point, then that first point was considered as peak onset. Peak amplitude was calculated by subtracting the voltage value at onset from the maximal value. This process was repeated 10,000 times to create a population of predicted amplitudes based of T-only and C-only traces (Fig. 1 *D* and *E*).

To quantify the modulatory effect of the C_stim_, the amplitudes of the MEP obtained with paired stimulation conditions were compared with the population of predicted amplitudes. For each of the 7 ISIs, the single trials (*n* = 100) were averaged and the MEP peak amplitude calculated as described above. The conditioned MEP peak amplitude was compared with the population of predicted amplitudes to establish the direction of modulation (facilitation, inhibition, or no modulation) and the normalized strength of modulation (*Z*-score) using the following formula:

$$Z\text{-score}=\frac{\text{Amplitude with ISI(}n\text{)}-\text{mean amplitude of predicted population}}{\text{SD of the predicted population}}$$

where *n* is the value of the ISI (e.g., ISI0). A negative *Z*-score indicates a decrease of the MEP amplitude by the C_stim_ in comparison to the predictor, which we refer to as an “inhibition.” A positive Z-score indicates an increase of the MEP amplitudes by the C_stim_ in comparison to the predictor, which refer to as a “facilitation.” Modulation was considered significant when the *Z*-score was greater than or equal to 1.96 (facilitation) or less than or equal to −1.96 (inhibition).

For each type of protocol (i.e., CFA-CFA, RFA-CFA, and RFA-RFA), we calculated the proportion of MEPs significantly modulated by the conditioning stimulus (i.e., incidence of significant effects) and the strength of the modulations (average *Z*-score). In addition, to provide a global measure that reflects the potential impact of CFA and RFA on motor outputs of the opposite hemisphere, we combined the incidence and magnitude values of modulatory effects into a single *impact score*, calculated for facilitatory or inhibitory effects separately. For each type of protocol, using data with all ISIs and all three muscles combined, we multiplied incidence of significant effects by the mean magnitude of these significant effects.

#### Statistical analysis.

We first examined the output properties of CFA and RFA using the MEPs with the T-only condition. A one-way ANOVA compared the absolute amplitudes of MEPs from the three types of interaction protocols, as well as to compare the latencies of MEPs from CFA across all three muscles and onset latencies of MEPs from RFA across all three muscles. Bonferroni corrected post hoc *t* tests were used if the main effect of ANOVA was significant. The stimulation intensity used for the T_stim_ in CFA and in RFA protocols and the mean onset latency of MEPs evoked from RFA and CFA were compared using two-sample Student *t*-tests.

Second, we examined the modulatory effects of the conditioning stimulation on MEPs with the various ISIs. Combining data from the three types of protocols, we used an ANOVA to test if the magnitudes of modulation of MEPs (*Z*-score) was different in the three muscles. Linear regressions were used to test if there was a relation between the magnitude of the modulation and onset latency of the MEPs with T-only stimulations. One regression was used for each type of protocol, and each regression combined data from all seven ISIs and all three muscles. The comparisons of incidence of facilitatory or inhibitory effects were performed with Chi-square (χ^2^)-tests, followed by a post hoc two-proportion *Z*-tests. Similarly, to compare the pattern of modulation across ISIs and the pattern of modulation across muscles evoked with the three types of protocols, χ^2^-tests followed by a post hoc two-proportion *Z*-tests were used. Comparisons of the magnitude of effects between the three types of protocols were performed with two one-way ANOVAs, one for facilitatory and one for inhibitory effects. Bonferroni post hoc tests were used if significant main effects were identified between protocols.

For all analyses using χ^2^-tests, a total of three tests were conducted: *1*) comparing CFA-CFA protocols with RFA-CFA protocols; *2*) comparing CFA-CFA with RFA-RFA protocols; and *3*) comparing RFA-CFA with RFA-RFA protocols. With the adjustment for multiple comparisons, *P* ≤ 0.017 was considered significant. For all other tests including the post hoc two-proportion *Z*-test, *P* ≤ 0.05 was considered significant. Unless otherwise specified, results are expressed as means ± SE. All statistical analyses were performed using MATLAB (Version R2014a).

## RESULTS

Using the data obtained with the T-only stimulation, we first characterized the output properties of responses from CFA and RFA in the arm contralateral to T_stim_ (Fig. 1*A*). For protocols testing the modulatory effects from CFA on the motor outputs of its homolog (CFA-CFA protocols), we obtained 60 significant MEPs (>1 SD above baseline; see materials and methods) with T-only stimulation (20 in WE, 20 in WF and 20 in EF). For protocols testing modulatory effects from RFA on motor outputs of CFA in the opposite hemisphere (RFA-CFA protocols), we found 49 significant MEPs with T-only stimulation (17 in WE, 16 in WF, and 16 in EF). Finally, in protocols that tested the modulatory effects from RFA on motor outputs of its homolog (RFA-RFA protocols), we found 34 significant MEPs with T-only stimulation (13 in WE, 9 in WF, and 12 in EF). Then, we studied the effects of C_stim_ delivered in the opposite hemisphere with different ISIs on these motor outputs (Fig. 1, *D* and *E*).

### 

#### Comparison of output properties of CFA and RFA.

**Fig. 2.**
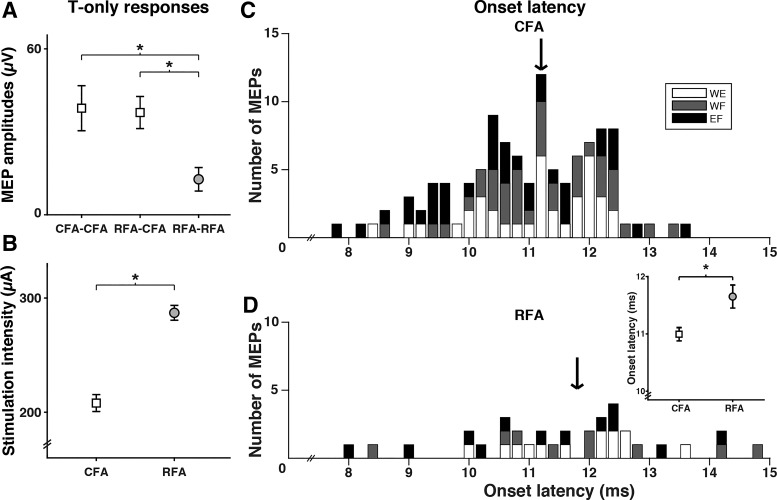
Comparison of output latencies from caudal and rostral forelimb areas (CFA and RFA). *A*: mean peak amplitude (±SE) of motor-evoked potentials (MEPs) in the 3 types of protocols (CFA-CFA, RFA-CFA, and RFA-RFA). MEPs from CFA, regardless if they were collected in the CFA-CFA or RFA-CFA protocols, had similar amplitudes. They were however significantly greater than MEPs from RFA. *B*: mean threshold stimulation intensity (±SE) necessary to evoke responses from CFA and RFA. Threshold current intensity values were significantly greater in RFA than in CFA. *C*: MEP onset latency resulting from test stimulus delivered alone (T-only) stimulation in CFA (*n* = 109) of all 3 muscles [wrist extensor (WE): white; wrist flexor (WF): gray; elbow flexor (EF)]. The histogram shows the count of MEPs with different onset latency values (bins of 0.2 ms). Onset latencies of MEPs induced by CFA ranged from 7.8 ms to 13.7 ms, with a median value of 11.2 ms (black arrow). No clear differences of onset latencies were observed between muscles. *D*: onset latencies of MEPs resulting from T-only stimulation in RFA (*n* = 34). Onset latencies of MEPs induced by RFA ranged from 8.0 ms to 14.7 ms, with a median value of 11.8 ms (black arrow). Again, no clear differences for the 3 muscles tested were observed. *Inset*: average (±SE) of onset latencies of MEPs from CFA was shorter than from RFA. *P < 0.05.

Using the significant MEPs (>1 SD above baseline) with T-only stimulation condition (mean current intensity ± SD = 253 ± 7.1 µA; max = 300 µA), we wanted to establish whether the output properties of CFA and RFA were different. We compared the amplitudes (µV) of MEPs with the T-only condition in the three types of protocols (Fig. 2*A*) and found significant differences [*F*_(2,140)_ = 3.41, *P* = 0.04]. The amplitudes of MEPs from RFA (RFA-RFA protocols means ± SE = 13.6 ± 4.2 µV) were significantly smaller than the ones from CFA (CFA-CFA: 38.9 ± 8.0 µV, *P* = 0.02; RFA-CFA: 37.4 ± 5.7 µV, *P* = 0.03). As expected, there was no significant difference in absolute amplitudes of MEPs evoked from CFA, whether the protocol was designed to test the modulatory effects of CFA or RFA (*P* = 0.9). Then, we compared the mean threshold T_stim_ intensities used in CFA (244.3 ± 12.5 µA; data from CFA-CFA and RFA-CFA protocols pooled) and RFA (287.1 ± 9.7 µA) and found that values for CFA were significantly lower [T_(49)_ = −2.02, *P* = 0.049] (Fig. 2*B*). Thus MEPs from RFA had smaller peak amplitudes than the ones from CFA, even though current intensities used to evoke these responses were greater. These results support that higher stimulation intensities are required to evoke MEPs from RFA in comparison to CFA.

We also verified if MEPs from CFA and RFA had different onset latencies. Once again, there was no difference of onset latencies for MEPs from CFA, whether they were collected during CFA-CFA or RFA-CFA protocols [11.0 ± 0.2 ms and 11.0 ± 0.2 ms, respectively; T_(107)_ = −0.18, *P* = 0.9]. We thus combined these data to obtain 109 MEPs with T-only stimulation in CFA. Figure 2*C* shows onset latencies of these 109 MEPs, and Fig. 2*D* shows onset latencies value of the 34 MEPs with T-only stimulation in RFA (RFA-RFA protocols). The range of latency values was greater for MEPs from RFA (range = 6.7 ms) than from CFA (range = 5.9 ms). For RFA, however, short latency responses (<10 ms) were less common (RFA = 8.8% vs. CFA = 17.4%) and long latency responses (>13 ms) more common (RFA = 14.7% vs. CFA = 2.7%). When comparing the mean onset latency of MEPs from CFA (11.0 ± 0.2 ms) to the one from RFA (11.6 ± 0.3 ms), we found it was significantly shorter [T_(141)_ = −2.56, *P* < 0.01]. We found no difference of onset latencies across all three muscles (WE, WF, and EF), regardless if the MEPs were from CFA [*F*_(2,106)_ = 2.74, *P* = 0.07] or RFA [*F*_(2,31)_ = 0.54, *P* = 0.6].

Together our analyses of MEPs amplitudes and latencies support the idea that the output properties of CFA and RFA are different, with RFA evoking smaller and slightly slower EMG responses than CFA.

#### Modulatory effects of CFA and RFA with each ISI tested.

**Fig. 3.**
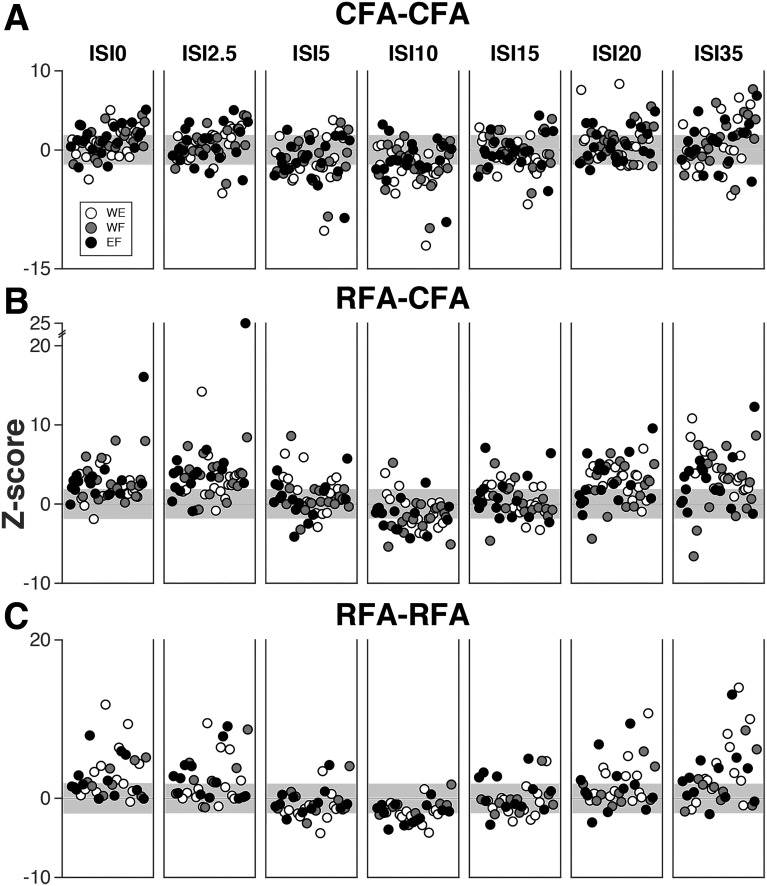
Complete data set of modulatory effects from caudal and rostral forelimb areas (CFA and RFA) on outputs from the opposite hemisphere. *A*: magnitude of modulatory effects for CFA-CFA protocols. The graph combines data from all rats and all 3 muscles [wrist extensor (WE): white; wrist flexor (WF): gray; elbow flexor (EF)]. Each dot reports the magnitude of the modulation (*Z*-score) of 1 motor-evoked potential (MEP) by the conditioning stimulation in CFA. Dots are ordered based on the onset latency of the MEP resulting from test stimulus (T_stim_) delivered alone (T-only) stimulation (increasing values from *left* to *right*) and the order is kept constant across panels showing modulations with the different interstimulus intervals (ISIs) [delays between the conditioning stimulus (C_stim_) and T_stim_]. For this type of protocol, there were 60 significant MEPs (> 1 SD above baseline) with the T-only condition in CFA (i.e., 60 circles plotted per ISI). With ISI0 for example (*leftmost panel*), out of these 60 MEPs 47 had larger values (above 0 in the plot), and 13 had smaller values (below 0 in the plot) with the conditioning stimulation. However, only 21 MEPs were significantly facilitated and 3 significantly inhibited (i.e., outside of the ± 1.96 *Z*-score range; gray background). *B*: magnitude of modulatory effects for RFA-CFA protocols presented as in *A*. In comparison with CFA, RFA appeared to induce more facilitatory effects on the outputs of CFA in the opposite hemisphere. *C*: magnitude of modulatory effects for RFA-RFA protocols presented as in *A*. There was no clear difference in the pattern of modulation between the muscles recorded.

For any given protocol, we obtained the population of predicted amplitudes evoked in the T-only and C-only trials (see materials and methods). We then compared the MEPs obtained in the paired-pulse conditions with each ISI to the population of predicted amplitudes. Figure 3 shows the complete data set of modulatory effects across rats with the different ISIs tested. The data are separated according to the three types of interaction protocols, respectively, testing the modulatory effects from CFA on the outputs of its homolog (CFA-CFA protocols, *n* = 20; Fig. 3*A*), from RFA on the outputs of CFA in the opposite hemisphere (RFA-CFA protocols, *n* = 17; Fig. 3*B*) and from RFA on the outputs of its homolog (RFA-RFA protocols, *n* = 14; Fig. 3*C*). For all three types of protocols, modulatory effects were clearly affected by ISIs. There were some common features observed for the three types of protocols. While more facilitatory effects were evoked with short (ISI0 and ISI2.5) and long ISIs (ISI20 and ISI35), inhibitory effects were most common with intermediate ISIs, in particular ISI10. There were also some clear differences between the three types of protocols. For example, inhibitory effects appeared to be more common and powerful for CFA-CFA protocols then for the others two types of protocols. In contrast, conditioning stimulations in RFA with short or long ISIs seemed to induce more powerful facilitatory effects than conditioning stimulation in CFA. Finally, Fig. 3, *A*–*C*, also shows no obvious differences in the pattern of modulation for the different muscles. This was confirmed statistically, as we found no difference in the magnitude of modulations of MEPs in the three muscles, when combining data from the three types of protocols [*F*_(2,998)_ = 0.34, *P* = 0.7]. Finally, combining data from the three muscles and seven ISIs, we found a weak correlation between the onset latency of MEPs with T-only stimulation and the magnitude of the modulation (*Z*-score) for CFA-CFA (rho = 0.156, *P* = 0.001) and RFA-RFA protocols (rho = 0.166, *P* = 0.01). This suggests that slower MEPs from CFA or RFA tended to be more facilitated when conditioning stimulation was delivered in the homolog cortical region. However, since this relation was weak, we did not investigate this possibility further.

**Fig. 4.**
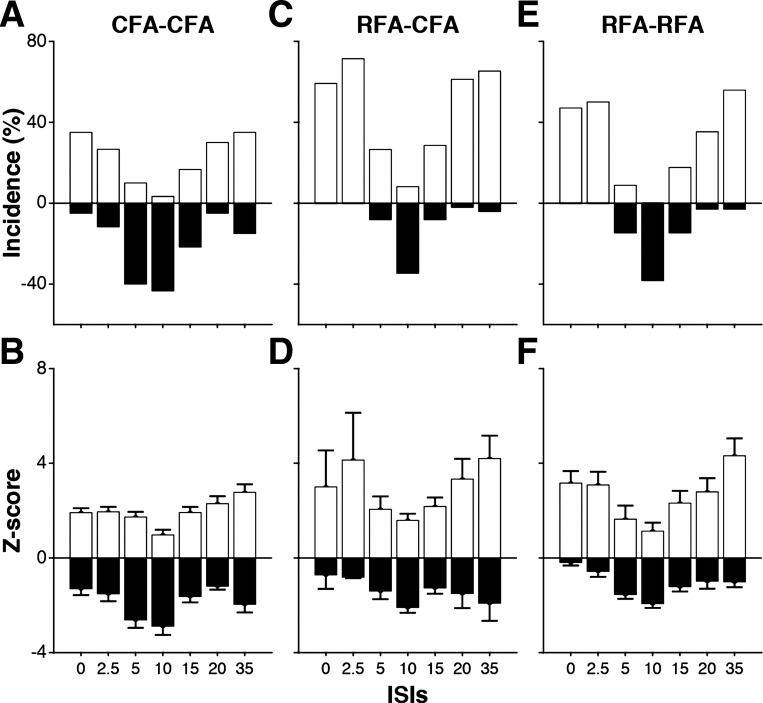
Quantification of modulatory effects of caudal and rostral forelimb areas (CFA and RFA) with each interstimulus interval (ISI) tested. *A*: for each ISI, the bars show the incidence (%) of significant modulation from CFA on the outputs of its homolog (CFA-CFA protocols; all 3 muscles combined). For each ISI, we calculated the proportion of significant facilitatory (white bars) and inhibitory (black bars) effects. With ISI0 for example, out of 60 motor-evoked potentials (MEPs) from CFA, 21 were significantly facilitated (35.0%), and 3 were significantly inhibited (3.5%). *B*: mean magnitude (*Z*-score ± SE) of facilitatory and inhibitory modulations from CFA on the outputs of its homolog (all 3 muscles combined). *C*: incidence of significant modulations from RFA on the outputs of CFA in the opposite hemisphere. Results are presented as in *A*. *D*: mean magnitude (±SE) of facilitatory and inhibitory modulations from RFA on the outputs of CFA in the opposite hemisphere. Results are presented as in *B*. *E*: incidence of significant modulations resulting from RFA on the outputs of its homolog in the opposite hemisphere. Results are presented as in *A*. *F*: mean magnitude (±SE) of facilitatory and inhibitory modulations resulting from RFA on the outputs of its homolog. Results are presented as in *B*.

For each ISI, we calculated the proportion of significant facilitatory and inhibitory effects induced by the conditioning stimulation (i.e., the numbers of MEPs with a *Z*-score ≥1.96 and ≤-1.96, respectively; see materials and methods) and the average magnitude of modulatory effects (mean *Z*-scores ± SE). For CFA-CFA protocols, out of the 420 MEPs (60 significant responses with T-only, conditioned with 7 ISIs), we found 179 (42.6%) cases in which CFA conditioning significantly modulated the outputs of its homolog. Out of these significant effects, there was a comparable number of facilitation (*n* = 94, 22.4%) and inhibition (*n* = 85, 20.2%). While both facilitatory and inhibitory effects were observed with every ISI, the proportion of facilitatory and inhibitory effects induced by CFA was highly variable with each ISI tested (Fig. 4*A*). Significant facilitatory effects were most common when the C_stim_ and T_stim_ were delivered simultaneously (ISI0: *n* = 21, 35.0%) or separated with 35 ms (ISI35: *n* = 21, 35.0%). In contrast, significant inhibitory effects were most common with intermediate ISIs (ISI5: *n* = 24, 40.0%; and ISI10: *n* = 26, 43.3%). Figure 4*B* shows the average magnitude of modulations induced by CFA on the outputs of its homolog with each ISI. Note that to better reflect the general magnitude of modulations, these data include all MEPs (see Fig. 2) and are not restricted to the MEPs that were significantly modulated. In general, the magnitude of modulatory effects from CFA followed a similar pattern with the various ISIs as described for the incidence. The strongest facilitatory effects were observed when the C_stim_ was delivered at the same time or shortly before the T_stim_ (i.e., ISI0 and ISI2.5) or with long ISIs (i.e., ISI20 and ISI35). Inhibition was most powerful with intermediate ISIs, in particular with ISI5 and ISI10.

For RFA-CFA protocols, out of the 343 MEPs (49 significant responses with T-only conditioned with 7 ISIs), we found 185 (53.9%) cases in which RFA conditioning significantly modulated the outputs of CFA in the opposite hemisphere. Out of these significant effects, there were many more cases of facilitation (*n* = 157, 45.7%) than cases of inhibition (*n* = 28, 8.2%) (Fig. 4*C*). As described for CFA-CFA protocols, significant facilitatory effects were most common with short (ISI0: *n* = 29, 60.0%; ISI2.5: *n* = 35, 71.4) or long ISIs (ISI20: *n* = 30, 61.2%; and ISI35: *n* = 32, 65.3%) and significant inhibitory effects were most common with intermediate ISIs, in particular ISI10 (*n* = 17, 34.7%). In fact, this was the only ISI with which inhibitory effects were more common than facilitatory effects. Furthermore, conditioning of RFA did not induce any case of inhibition with short ISIs (ISI0 and ISI2.5). For the magnitude of facilitatory effects (Fig. 4*D*), the most powerful facilitatory effects were observed with short (ISI0 and ISI2.5) and long ISIs (ISI20 and ISI35). Inhibition was more powerful with ISI10 but also when longer delays were used between the conditioning and test stimuli (ISI20–ISI35).

For RFA-RFA protocols, from the 238 MEPs (34 significant responses with T-only conditioned with 7 ISIs), we found 98 cases in which RFA conditioning significantly modulated the outputs of its homolog (41.2%). Again, for this type of protocol, RFA conditioning induced many more cases of facilitation (*n* = 73, 30.7%) than inhibition (*n* = 25, 10.5%) (Fig. 4*E*). Facilitatory effects were most common with short (ISI0: *n* = 16, 47.1%; ISI2.5: *n* = 17, 50.0%) and long ISIs (ISI20; *n* = 12, 35.3%; ISI35: *n* = 19, 55.9%), and inhibitory effects were most common with intermediate ISIs (ISI5: *n* = 5, 14.7%; ISI10: *n* = 13, 38.2%; and ISI15: *n* = 5, 14.7%). As for RFA-CFA protocols, and in contrast to CFA-CFA protocols, conditioning stimulations exclusively induced facilitatory effects with ISI0 and ISI2.5. For both the facilitatory and inhibitory effects, the magnitude of modulation with the different ISIs followed a similar pattern as the one described for incidence (Fig. 4*F*). The strongest facilitatory effects were observed when the C_stim_ was delivered at the same time or shortly before the T_stim_ (i.e., ISI0 and ISI2.5) or with long ISIs (i.e., ISI20 and ISI35). Inhibition was most powerful with intermediate ISIs, in particular with ISI5 and ISI10.

In summary, these figures highlight that there are clear similarities in the pattern of modulatory effects evoked with the various ISIs for the three interaction protocols studied. Cortical motor areas of the contralateral hemisphere (i.e., both CFA and RFA) induce more frequent and powerful facilitation of motor outputs with short (ISI0 and ISI2.5) and long ISIs (ISI20 and ISI35). In contrast, frequent and powerful inhibitory effects are induced when conditioning is delivered 10 ms before the T_stim_. These figures also reveal clear differences between the three types of protocols. They show that CFA can induce inhibitory effects with all the ISIs we tested and that inhibitory effects are not only frequent and powerful with ISI10 but also with ISI5. In contrast, RFA exclusively induces facilitatory effects when the C_stim_ is delivered simultaneously (ISI0) or 2.5 ms prior (ISI2.5) to the T_stim_ and that regardless if it is to modulate outputs of CFA or RFA in the opposite hemisphere.

#### Comparison of the modulatory effects of CFA and RFA with all ISIs combined.

**Fig. 5.**
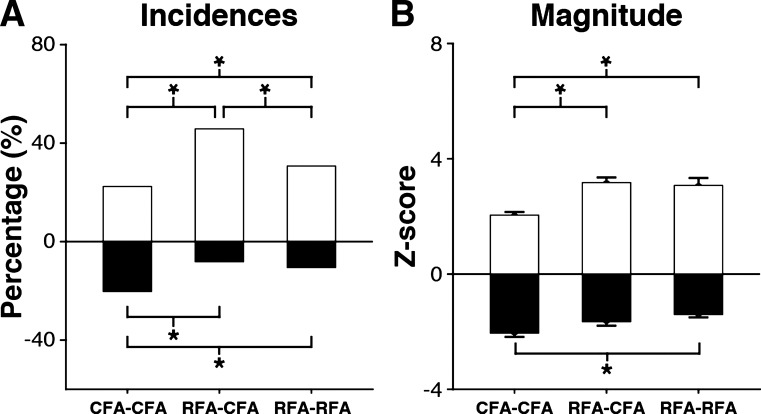
Comparison of the modulatory effects of caudal and rostral forelimb areas (CFA and RFA) with all interstimulus intervals (ISIs) combined. *A*: comparison of the incidence of modulatory effects for CFA-CFA, RFA-CFA, and RFA-RFA protocols with all the ISIs tested combined. We found significantly fewer facilitatory effects and more inhibitory effects for CFA-CFA protocols than for the other 2 types of protocols. In addition, RFA-CFA induced more facilitatory effects in comparison to RFA-RFA protocols. *P ≤ 0.017. *B*: comparison of the magnitude of modulatory effects from all 3 protocols with all the tested ISIs combined. We found that facilitatory effects of CFA-CFA protocols were significantly weaker than for other protocols. Inhibitory effects were significantly more powerful for CFA-CFA than RFA-RFA protocols. *P ≤ 0.05.

To statistically compare the overall incidence of modulatory effects for the three types of protocols, we pooled the significant effects evoked with all ISIs (Fig. 5*A*). First, we wanted to know if delivering the C_stim_ in CFA versus RFA induced different proportions of facilitatory and inhibitory effects on the outputs of CFA in the opposite hemisphere (i.e., CFA-CFA vs. RFA-CFA protocols) and found it did (χ^2^ = 54.6; *P* < 0.001). CFA conditioning induced less facilitation (*n* = 94, 22.4%) than RFA conditioning (*n* = 157, 45.8%; *P* < 0.001) and more inhibition (CFA *n* = 85, 20.2% vs. RFA *n* = 28, 8.2%; *P* < 0.001) on the outputs of CFA in the opposite hemisphere. Second, we asked if CFA and RFA induced different proportions of facilitatory and inhibitory effects on the outputs of their homologs (i.e., CFA-CFA versus RFA-RFA protocols). The incidence of significant effects was different (χ^2^ = 12.8; *P* = 0.01), with CFA inducing fewer facilitatory effects (*n* = 94, 22.4%) than RFA (*n* = 73, 30.7%; *P* = 0.02) and more inhibitory effects (*n* = 85, 20.2%) than RFA (*n* = 25, 10.5%; *P* = 0.001). Finally, we compared the proportions of facilitatory and inhibitory effects induced by RFA on the outputs of CFA and RFA in the opposite hemisphere (i.e., RFA-CFA versus RFA-RFA protocols) and found it was different (χ^2^ = 13.4; *P* = 0.01). The incidence of facilitatory effects induced by RFA was greater on the outputs of CFA (RFA-CFA: *n* = 157, 45.8%) than on the output of RFA (RFA-RFA: *n* = 73, 30.7%; *P* < 0.001), but the incidence of inhibitory effects was comparable (RFA-CFA: *n* = 28, 8.2%; RFA-RFA: *n* = 25, 10.5%, *P* = 0.3).

The magnitude of modulatory effects evoked with the various ISIs were also pooled to compare the three types of protocols (Fig. 5*B*). The magnitude of facilitatory effects was different [*F*_(2,594)_ = 13.5; *P* < 0.001]. Facilitatory effects from CFA on the outputs of its homolog were weaker (CFA-CFA: *Z*-score = 2.05 ± 0.11) than the ones from RFA. This was true for both when RFA modulated the outputs of CFA in the opposite hemisphere (RFA-CFA: *Z*-score = 3.17 ± 0.18, *P* < 0.001), or of its homolog (RFA-RFA: *Z*-score = 3.07 ± 0.26, *P* < 0.001). However, the magnitude of facilitatory effects from RFA on the outputs of its homolog and of CFA in the opposite hemisphere were not significantly different (*P* = 1.0). For the magnitude of inhibitory effects, there were also significant differences for the three types of protocols [*F*_(2,401)_ = 6.1, *P* = 0.002]. CFA induced more powerful inhibition on the outputs of its homolog (CFA-CFA: *Z*-score = −2.05 ± 0.13) than RFA on the outputs of its homolog (RFA-RFA: *Z*-score = −1.40 ± 0.01; *P* = 0.003).

In conclusion, while the conditioning of CFA induced fewer and weaker facilitatory effects, it induced more and generally stronger inhibitory effects than the conditioning of RFA. For RFA conditioning, the magnitude of modulations was similar on both the motor output of CFA or of its homolog in the opposite hemisphere. However, facilitatory effects were more common when RFA modulated the outputs of CFA than the outputs of its homolog in the opposite hemisphere.

#### Pattern of modulatory effects from CFA and RFA across ISIs.

Next, we characterized how individual MEPs were modulated across the seven ISIs tested and compared the pattern of modulations for the three types of protocols. Out of the significant MEPs evoked with the T-only condition (>1 SD above baseline), we found few that were not modulated with any of the ISIs tested for CFA-CFA (8 out of 60 MEPs; 13.3%; number cortical sites tested or protocols = 20), RFA-CFA (2 out of 49 MEPs; 4.1%; number of protocols = 17), and RFA-RFA (4 out of 34 MEPs; 11.8%; number of protocols = 14). This supports that the conditioning stimulations in either CFA or RFA were very likely to affect motor outputs of the opposite hemisphere with one or more of the ISIs tested. For these modulated MEPs, we separated the pattern of modulation across ISIs into three groups (Deffeyes et al. 2015). First, for a given MEP, the conditioning stimulation could be significantly facilitatory with one or more ISIs and never induce significant inhibition across the ISIs tested (i.e., group pure facilitation). Second, the conditioning stimulation could be significantly inhibitory with one or more ISIs and never induce significant facilitation across the ISIs tested (i.e., group pure inhibition). Third, the conditioning stimulation could be significantly facilitatory with one or more ISIs and significantly inhibitory one or more ISIs (i.e., group opposite).

**Fig. 6.**
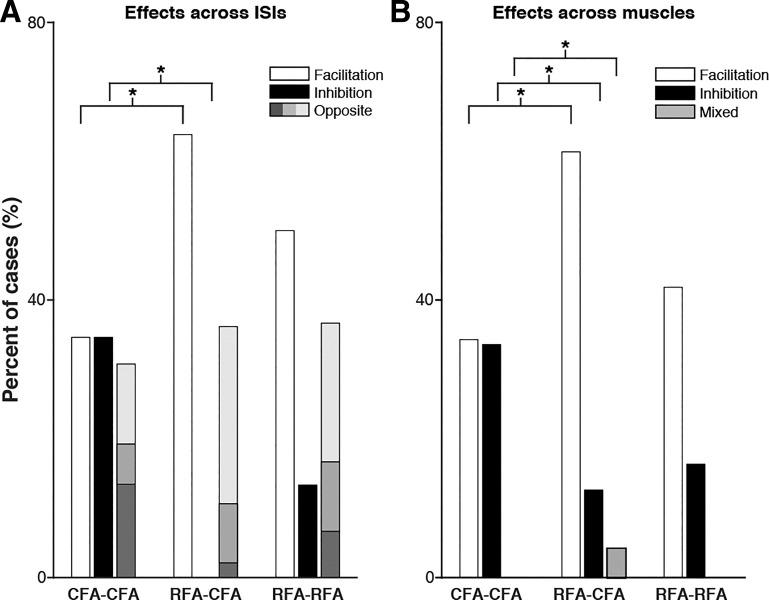
Pattern of modulatory effects from caudal and rostral forelimb areas (CFA and RFA) across interstimulus intervals (ISIs) and across muscles. *A*: the bar graph reports the incidence of modulatory effects across ISIs for the 3 types of protocols. For CFA-CFA protocols, we found comparable proportions of pure facilitatory (white bar), pure inhibitory (black bar), and opposite effects (gray bar) across ISIs. Within the population of opposite effects, there were fewer cases with an equal number of facilitation and inhibition across ISIs (mid-tone gray) than cases with a predominance of facilitation (light gray) or a predominance inhibition across ISIs (dark gray). For RFA-CFA protocols, we found more cases of pure facilitation and no cases of pure inhibition. Proportions of pure facilitation and inhibition differed significantly between CFA-CFA and RFA-CFA protocols. In addition, there were many more cases of opposite effects with a predominance of facilitatory effects across ISIs (light gray). For RFA-RFA protocols, the pattern of modulation across ISIs was somewhat in between the 2 other types of protocols and there was no difference in the pattern of modulation across ISIs in comparison to the other types of protocols. *P ≤ 0.017. *B*: incidence of modulatory effects across muscles for the 3 types of protocols presented as in *A*. Proportion of pure facilitation across muscles (white bars), pure inhibition (black bars), and mixed effects across muscles (gray). For CFA-CFA protocols, we found comparable proportion of cases of pure facilitation and pure inhibition, and no cases of mixed effects. In contrast, conditioning stimulation in RFA evoked many more pure facilitation effects across muscles than pure inhibition or mixed effects, and this was true for both the outputs of CFA or RFA in the opposite hemisphere. In all cases of mixed effects in RFA-CFA protocols, there were an equal number of muscles simultaneously facilitated and inhibited (mid-tone gray). When comparing the 3 types of protocols, we found that CFA-CFA protocols evoked fewer pure facilitation and mixed responses than RFA-CFA protocols, and more inhibition than RFA-CFA protocols. There was no difference in the pattern of modulation across muscles from RFA on the outputs of either CFA or RFA in the opposite hemisphere (RFA-CFA vs. RFA-RFA). *P ≤ 0.017.

For CFA-CFA protocols, out of the 52 MEPs that were significantly modulated with at least 1 ISI, CFA conditioning induced a comparable number of cases of pure facilitation (*n* = 18; 34.6%), pure inhibition (*n* = 18; 34.6%), and opposite effects (*n* = 16; 30.7%) (Fig. 6*A*). The pattern of modulation of CFA outputs across ISIs was very different when the conditioning stimulation was applied in RFA (RFA-CFA protocols; χ^2^ = 20.71, *P* < 0.001). Out of 47 that were significantly modulated with at least 1 ISI, most cases were pure facilitation (*n* = 30; 63.8%), some were opposite (*n* = 17; 36.2%) and none were pure inhibition. Thus all cortical sites tested in RFA that modulated the outputs of CFA induced significant facilitation with at least one of the ISI tested. When comparing the patterns of modulation of these two types of protocols, we found that conditioning stimulation delivered in CFA evoked significantly fewer cases of pure facilitation (*P* = 0.004) and more cases of pure inhibition (*P* < 0.001) than when the conditioning stimulations were delivered in RFA. When RFA conditioned the outputs of its homolog (i.e., RFA-RFA protocols), values lied somewhat in between the ones of the other two types of protocols. Out of 30 MEPs significantly modulated with at least 1 ISI, cases of pure facilitation (*n* = 15; 50.0%) were more common than opposite effects (*n* = 11; 36.7%), and cases of pure inhibition were rarely observed (*n* = 4; 13.3%). These values were not significantly different than the ones of CFA-CFA (χ^2^ = 7.6, *P* = 0.03) or RFA-CFA (χ^2^ = 5.61, *P* = 0.06) protocols.

#### Pattern of modulatory effects from CFA and RFA simultaneously induced across muscles.

We then studied how a given cortical site in CFA or RFA simultaneously modulated the outputs to various muscles. Once again, we separated patterns of modulation into three different groups. First, a given cortical site in CFA or RFA could induce a significant facilitation on the MEP of one and up to all three muscles simultaneously (i.e., group pure facilitation across muscles). Second, it could induce a significant inhibition on the MEP of one and up to all three muscles (i.e., group pure inhibition across muscles). Third, the conditioning stimulation could simultaneously facilitate and inhibit different combinations of muscles (i.e., group simultaneous mixed effects across muscles). For a given protocol, we calculated the proportion of occurrence of these groups of effects across muscles for all seven ISIs.

For protocols testing the modulatory effects from CFA on the outputs of its homolog (CFA-CFA protocols; 20 cortical sites tested × 7 ISIs = 140 cases), we found 48 cases (34.3%) of pure facilitation and a comparable proportion of pure inhibition across muscles (*n* = 47; 33.6%) (Fig. 6*B*). Out of these cases, 29 (20.7%) involved simultaneous facilitation in more than 1 muscle and 26 (18.6%) simultaneous inhibition in more than 1 muscle. We found no case in which conditioning stimulation in CFA simultaneously facilitated the outputs to one muscle while inhibiting outputs to another muscles (i.e., mixed effects). The pattern of modulation across muscles was very different for protocols testing the modulatory effects from RFA on the outputs of CFA in the opposite hemisphere (RFA-CFA protocols; 17 protocols × 7 ISIs = 119 cases; χ^2^ = 24.4; *P* = 0.001). There were many more cases of pure facilitation across muscles (*n* = 73; 61.3%) than pure inhibition (*n* = 15; 12.6%). Out of these cases, 49 (35.0%) involved simultaneous facilitation in more than 1 muscle and 5 (3.6%) simultaneous inhibition in more than 1 muscle. In addition, we found a few cases in which RFA simultaneously induced facilitation and inhibition in different muscles (*n* = 5; 4.2%). When comparing the patterns of modulation for the two types of protocols, we found that conditioning stimulation delivered in CFA evoked significantly less pure facilitatory (*P* < 0.001) and mixed effects (*P* = 0.01) but significantly more pure inhibitory effects (*P* < 0.001). Once again, the pattern of modulatory effects for protocols testing the effects from RFA on the outputs of its homolog was somewhat in between the ones of CFA-CFA (χ^2^ = 8,8, *P* = 0.6) and RFA-CFA (χ^2^ = 11.2, *P* = 0.2) protocols, with no significant differences. Out of the 98 cases (14 protocols × 7 ISIs), we found many more pure facilitation (*n* = 41; 41.8%) than pure inhibition (*n* = 16; 16.3%) across muscles and no mixed effects. Out of these cases, 22 (15.7%) involved simultaneous facilitation in more than one muscle and 6 (4.3%) simultaneous inhibition in more than 1 muscle.

#### Comparison of global modulatory impact of CFA and RFA.

**Fig. 7.**
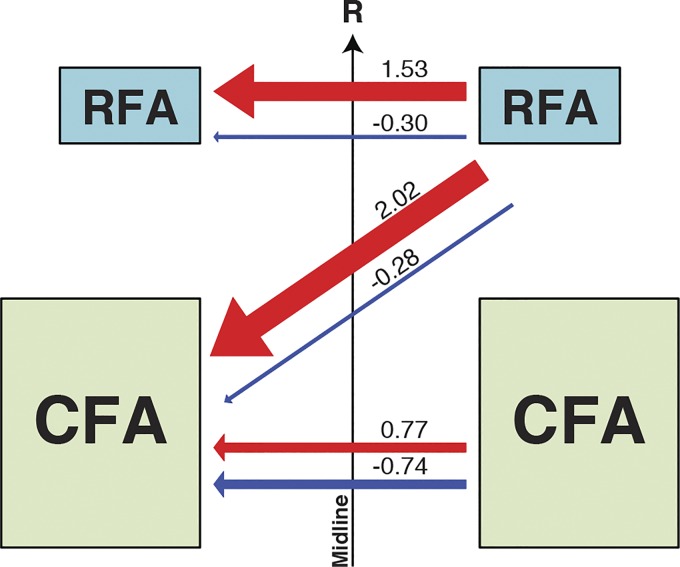
Global impact scores of facilitatory and inhibitory effects of caudal and rostral forelimb areas (CFA and RFA) on motor outputs. Box and arrow diagram summarizing the impact scores of CFA and RFA on motor outputs from the opposite hemisphere. The numbers next to each arrow report the impact score value and the thickness of the arrow is proportional to this value. Impact scores were calculated by multiplying the incidence by the mean of all significant modulations. For example, for all CFA-CFA protocols with all interstimulus intervals (ISIs) combined, the ratio of significant facilitatory effects was 0.224 (22.4%) and the average *Z*-score of these significant effects was 3.42. Therefore, the facilitatory impact factor of CFA on the output of its homolog was 0.77. For CFA-CFA, the impact scores for facilitatory and inhibitory effects were quite similar, suggesting CFA has comparable capacity to facilitate or inhibit the outputs of its homolog. In contrast for both RFA-CFA and RFA-RFA, the impact scores for facilitatory effects were much greater than for inhibitory effects. This suggests that RFA has a greater potential to facilitate motor outputs from the opposite hemisphere, regardless if the output is originating from CFA or RFA. Overall, the greatest facilitatory impact was from RFA on the outputs of its homolog and the greatest inhibitory impact was from CFA on the outputs of its homolog.

In a last series of analyses, we wanted to provide a “global” measure that reflects the potential impact from CFA and RFA on motor outputs of the opposite hemisphere. To do so, for each type of protocol we combined the incidence of significant modulations and their magnitude values into a single *impact score* for facilitation and one for inhibition (see materials and methods; Fig. 7). The greatest facilitatory impact score was from RFA on the outputs of CFA in the opposite hemisphere (2.02). This value was much larger than the facilitatory impact of RFA on its homolog (1.53) and more than twice the facilitatory impact of CFA on its homolog (0.77). In contrast, the greatest inhibitory impact score was from CFA on the output of its homolog (−0.74). This value was more than twice the inhibitory impact of RFA conditioning on either the outputs of its homolog or of CFA (−0.30 and −0.28, respectively). When looking at the pattern of modulation for each type of protocols, it appears that CFA has a balanced pattern of modulation, with a comparable capacity to facilitate or inhibit the outputs of its homolog in the opposite hemisphere. In contrast, RFA has a much greater potential to facilitate motor outputs from the opposite hemisphere, regardless if the output is originating from CFA of the opposite hemisphere or from its homolog.

## DISCUSSION

In the present study, we first characterized the output properties of CFA and RFA using single-pulse intracortical microstimulation techniques and found that CFA evokes larger EMG responses with slightly shorter latencies in comparison to RFA. Then, we studied how conditioning stimulation delivered in either CFA or RFA in the opposite hemisphere affected these outputs. Facilitatory effects from both CFA and RFA were always more frequent when the C_stim_ and T_stim_ were delivered with short or with long ISIs. In contrast, inhibitory effects were more common with intermediate ISIs. There were also differences between the modulatory effects induced by CFA and RFA. When the conditioning stimulation was delivered in CFA, inhibitory effects were more common than when it was in RFA. In contrast, RFA induced many more facilitatory effects and they were stronger than the ones from CFA. These data support that motor areas in rats can exert powerful and complex modulations of motor outputs from the opposite hemisphere. They also suggest that CFA and RFA make distinct contributions for the production of these outputs.

### 

#### Output properties of CFA and RFA.

Output properties of RFA and CFA have been compared in a previous electrophysiological study in albino rats (Liang et al. 1993), although in lightly sedated preparations with active EMG background. Under these conditions, EMG responses were evoked with much lower stimulation intensities and had shorter onset latencies than in our study. Furthermore, following stimulations in CFA and RFA, MEPs had similar latencies, although CFA was much more likely to evoke responses than RFA (1.5 to 3 times). The lower efficacy of RFA to evoke responses in forelimb muscles is thus a consistent finding across studies. With intensities based on threshold current values, we found that smaller currents were required to evoke responses from CFA and yet that these MEPs were greater than those from RFA. Similarly, in macaques, much larger responses are evoked from M1 than from premotor areas (Boudrias et al. 2010a, 2010b). Across premotor areas, stimulation in ventral premotor cortex evokes the largest responses, which are nonetheless more than seven times smaller than the ones from M1. Accordingly, although there is a clear difference in the efficiency of CFA and RFA to evoke responses in contralateral forelimb muscles, this difference is much smaller than the one between M1 and premotor areas in primates. The larger difference of output efficacy in primates may be due to the addition of more direct corticomotoneuronal projections from M1 onto cervical motoneurons (Rathelot and Strick 2006, 2009), a trait that appears to be lacking for CFA in rats (Alstermark et al. 2004; Yang and Lemon 2003).

The lower stimulation intensities and faster responses under light sedation (Liang et al. 1993) could be explained by higher summation probability of outputs from multiple neurons near firing threshold at the time of stimulation and the amplification of potential synchrony effects (Schieber and Rivlis 2005). This increased excitability of neurons in the various structures targeted by the outputs of CFA and RFA could also explain the presence of ipsilateral responses under light sedation (Liang et al. 1993), which we generally failed to find (only 3 out of 58 protocols; see materials and methods). Given our relatively limited data set for RFA (*n* = 34) and the methodological differences between our study and previous work, the potential differences between the average output latency of MEPs from CFA and RFA will need further investigation.

#### Similarities of modulatory effects from CFA and RFA.

For both CFA and RFA, using very short (0 and 2.5 ms) or long delays (20 and 35 ms) between the C_stim_ and T_stim_ evoked many more facilitatory than inhibitory effects. With long delays, the potential locus of interactions and pathways involved are numerous. They may favor effects carried by slower conducting fibers, oligosynaptic pathways between the two hemispheres, or give time for outputs from CFA or RFA to induce changes of excitability at downstream sites of convergence with outputs from the opposite motor areas. There are fewer mechanisms that can sustain modulations with very short delays. Considering that callosal conduction takes at least 5 ms in adult rats (Seggie and Berry 1972), it is unlikely that these very fast interactions take place at the cortical level. It is more probable that descending outputs from the two hemispheres interact at converging sites along the motor axis, in particular if we assume that conduction velocities of outputs from the two stimulation sites are comparable. Uncrossed corticospinal projections could be involved, although they are not numerous in rats (Brösamle and Schwab 1997, 2000). An alternative pathway is through cortical projections to the red nucleus, on either side of the brain. However, there are also few corticorubral projections that cross midline (Bernays et al. 1988; Naus et al. 1985; Z’Graggen et al. 1998). Rather, short latency interactions may primarily take place in the reticular formation, which does receive numerous projections from the ipsi and contralateral motor cortex (Shammah-Lagnado et al. 1987; Valverde 1962). Corticoreticular projections play a crucial role for the control of hand movements in rats (Alstermark and Pettersson 2014; Whishaw et al. 1998). The powerful short-latency facilitatory effects could thus favor efficacious integration of outputs from the two hemispheres for the execution of forelimb movements.

With intermediate ISIs (ISI5-ISI15), but particularly ISI10, conditioning stimulations in either CFA or RFA induced many more inhibitory than facilitatory effects. In adult rats, the onset of transcallosal cortical responses occur ~5 ms and peak 13 ms after stimulation (Seggie and Berry 1972). Based on these values, modulatory effects observed with ISI5 could potentially be due to callosal interactions, in particular if the callosal inputs affect the I waves created by the T_stim_ (Ziemann et al. 1998). However, using 10 ms of delay would provide sufficient time for more callosal inputs to reach the opposite cortex at the time when the T_stim_ was delivered and modulate its output. Because the vast majority (95–97%) of callosal neurons in rats are excitatory (Gonchar et al. 1995), transcallosal inhibition is likely due to intracortical microcircuitry at the postsynaptic site and connections onto GABAergic interneurons (Karayannis et al. 2007). This indirect pattern of connection may favor the greater incidence of inhibitory effects observed at ISI10 in comparison to ISI5. In contrast, direct excitatory callosal projections onto pyramidal neurons in deep cortical layers (Karayannis et al. 2007) could be involved in facilitatory effects observed with shorter ISIs (e.g., ISI2.5 and ISI5). Overall, the observed predominance of inhibitory effects at ISI5 and ISI10 aligns with findings in cats, in which callosal projections have also been reported to mainly exert inhibition on motor cortex (Asanuma and Okuda 1962). In rats, the release of callosal inhibition following cortical injury could explain the increased hemodynamic responses in the contralesional hemisphere (Dijkhuizen et al. 2001).

#### Differences of modulatory effects from CFA and RFA.

One main difference between the pattern of modulation of CFA and RFA is that the conditioning stimulation in CFA induced more powerful and a greater proportion of inhibitory effects than RFA. For both CFA and RFA, the primary target of interhemispheric projections is the homolog region in the opposite hemisphere (Rouiller et al. 1993). If inhibitory effects are mainly carried through callosal projections as discussed above, the number of connections could account for the more potent inhibitory effects from CFA on its homolog than from RFA on the outputs of CFA in the opposite hemisphere. This pattern of connectivity, however, cannot explain why RFA has weaker inhibitory effects on the outputs of its homolog than CFA on its homolog. We estimate that the radius of current spread with the stimulation intensities we used (i.e., ≤225 µA; radius ≤0.4 mm using a *k* value of 1,292 mA/mm^2^) (Stoney et al. 1968) should have been contained within both CFA or RFA, respectively, having surface areas of ~5.8 mm^2^ and ~1.2 mm^2^ in aged and sex-matched Sprague-Dawley rats (Touvykine et al. 2016). If the area of cortex directly activated by the C_stim_ was similar in both forelimb regions, one possibility is that the density of callosal neurons in CFA is greater than in RFA. Another possibility is that I waves evoked by the C_stim_ can propagate further in the larger CFA and eventually activate more callosal neurons that strengthen inhibitory effects observed with longer ISIs (15–35 ms).

A second major difference we found is that RFA induced more powerful and a greater proportion of facilitatory effects than CFA. As discussed above, short latency facilitatory effects may be largely carried through corticoreticular pathway, and, if so, our data suggest that these projections are much more impactful from RFA than from CFA. In NHPs, corticoreticular projections from premotor areas are denser than from M1 (Fregosi et al. 2017). If a similar pattern of projection for RFA and CFA exists in rats, it could explain how RFA can induce stronger short-latency facilitation. Although not as common, facilitatory effects from RFA on the outputs of its homolog were as strong as the ones from RFA on the outputs of CFA, and this was true for both short and long ISIs. It is not clear what is the role of such powerful facilitation between the two RFAs. However, it highlights the possibility that premotor areas in primates could dramatically affect their reciprocal outputs, a question that has received virtually no attention.

To date, studies on interhemispheric interactions in humans or NHPs have focused on the modulatory effects of premotor areas on M1 outputs (Buch et al. 2010; Côté et al. 2017; Fiori et al. 2017; Koch et al. 2007; Mochizuki et al. 2004a, 2004b; Quessy et al. 2016). In NHPs, we have compared the pattern of modulation from various premotor areas on M1 outputs using very similar techniques as the ones in the present study (Côté et al. 2017, 2020; Quessy et al. 2016). Much like RFA in rats, premotor areas induce strong modulations that are affected by the timing of stimulations. However, no single premotor area has a pattern of modulation that is similar to the one induced by RFA on the outputs of CFA in the opposite hemisphere. This suggests that the contribution of RFA to movement production is different from the one carried by the any one of the various premotor areas in primates. This is not particularly surprizing when considering that a common ancestor of rats and primates lived ~90–100 million years ago (Murphy et al. 2004; Nei et al. 2001), likely before the emergence of a “premotor” field (Kaas 2013). The development of these additional motor fields in rats and primates may thus be a phylogenetic convergence. They have emerged in parallel due to behavioral pressure to solve more complex motor problems but in a species-specific manner. Understanding these interspecies differences between rats and primates is important given the large use of rodent models in motor control and recovery studies. This knowledge can help us to make more accurate predictions of how the findings in rodents motor system can relate to mechanisms present in humans.

## GRANTS

This work was supported by the Natural Sciences and Engineering Research Council of Canada (NSERC Discovery Grant RGPIN-2016-05718 to N.D.). N.D. holds a Chercheur Sénior salary award from the Fonds de recherche du Québec–Santé (FRQS).

## DISCLOSURES

No conflicts of interest, financial or otherwise, are declared by the authors.

## AUTHOR CONTRIBUTIONS

N.D. conceived and designed research; B.T. performed experiments; B.T. and G.E. analyzed data; B.T., G.E., S.Q., and N.D. interpreted results of experiments; B.T. prepared figures; B.T. and N.D. drafted manuscript; B.T., G.E., S.Q., and N.D. edited and revised manuscript; B.T., G.E., S.Q., and N.D. approved final version of manuscript.
